# Anthropometric Study of Three-Dimensional Facial Morphology in Malay Adults

**DOI:** 10.1371/journal.pone.0164180

**Published:** 2016-10-05

**Authors:** Siti Adibah Othman, Lynnora Patrick Majawit, Wan Nurazreena Wan Hassan, Mang Chek Wey, Roziana Mohd Razi

**Affiliations:** 1 Department of Paediatric Dentistry and Orthodontics, University of Malaya, Kuala Lumpur, Malaysia; 2 Clinical Craniofacial Dentistry Research Group, Faculty of Dentistry, University of Malaya, Kuala Lumpur, Malaysia; Macquarie University, AUSTRALIA

## Abstract

**Objectives:**

To establish the three-dimensional (3D) facial soft tissue morphology of adult Malaysian subjects of the Malay ethnic group; and to determine the morphological differences between the genders, using a non-invasive stereo-photogrammetry 3D camera.

**Material and Methods:**

One hundred and nine subjects participated in this research, 54 Malay men and 55 Malay women, aged 20–30 years old with healthy BMI and with no adverse skeletal deviation. Twenty-three facial landmarks were identified on 3D facial images captured using a VECTRA M5-360 Head System (Canfield Scientific Inc, USA). Two angular, 3 ratio and 17 linear measurements were identified using Canfield Mirror imaging software. Intra- and inter-examiner reliability tests were carried out using 10 randomly selected images, analyzed using the intra-class correlation coefficient (ICC). Multivariate analysis of variance (MANOVA) was carried out to investigate morphologic differences between genders.

**Results:**

ICC scores were generally good for both intra-examiner (range 0.827–0.987) and inter-examiner reliability (range 0.700–0.983) tests. Generally, all facial measurements were larger in men than women, except the facial profile angle which was larger in women. Clinically significant gender dimorphisms existed in biocular width, nose height, nasal bridge length, face height and lower face height values (mean difference > 3mm). Clinical significance was set at 3mm.

**Conclusion:**

Facial soft tissue morphological values can be gathered efficiently and measured effectively from images captured by a non-invasive stereo-photogrammetry 3D camera. Adult men in Malaysia when compared to women had a wider distance between the eyes, a longer and more prominent nose and a longer face.

## Introduction

Anthropometry has been used in forensic science, for the purpose of understanding human physical variation, in paleoanthropology and in various attempts to correlate physical, ethnic and psychological traits. Nowadays, it plays an important role in industrial design, fashion design, ergonomics and architecture; where geometrical data about the distribution of body dimensions in the population are used to optimize product dimensions. Features distinguishing various ethnic groups were discovered when anthropometric methods were introduced into clinical practice; to quantify changes in the craniofacial framework. To successfully treat congenital or post-traumatic facial disfigurements in members of these groups, surgeons require access to craniofacial databases based on accurate anthropometric measurements. Normative data of facial measurements are indispensable to precisely determine the degree of deviation from normal [1]. Due to international migration in the contemporary world, it is important for professionals from various medical and dental specialities to be aware of differences in facial characteristics among ethnic groups; especially those whose work involves correction of facial anomalies and enhancing aesthetics [2].

Craniofacial anthropometric measurements require high quality measuring tools in order to get the highest possible measurement accuracy [3]. Currently, the most popular medical three-dimensional (3D) surface acquisition system is stereophotogrammetry [4]. Stereophotogrammetry offers many advantages over direct anthropometry. These advantages include speed of data collection, non-invasiveness, accurate 3D images and user friendliness. In addition, it has been proven to be reliable as a method to perform facial analysis; with the ability to obtain a 3D archive of a subject’s facial morphology [5–8]. Currently, 3D stereophotogrammetry imaging systems are more reliable, and hence have become the gold standard in facial anthropometry [9]. A systematic review done on the method to quantify soft-tissue also suggested that at present stereophotogrammetry seems to be the best 3D method for quantitative longitudinal assessment of facial dimensions in children [10].

Most anthropometric studies have indicated that normal measurements for one group should not be considered normal for other ethnic groups [1]. It is well established that human faces differ from one another based on ethnicities [1,11–17]. Hence, it is important to acquire anthropometric data for different ethnicities. Few studies have been undertaken on this subject. In China, soft tissue facial analysis of 50 men and 50 women was carried out using stereophotogrammetry to provide normative data of the Chinese face for surgeons [16]. Similar research was conducted by a Korean team studying normal occlusion of 30 men and 30 women between 21and 27 years old [12]. Stereophotogrammetry has been employed to study facial morphology in five other countries: Wales, United States, Hungary, Slovenia and Egypt [15].

An anthropometric study of the Malay ethnic group in Malaysia had been conducted by Ngeow and Aljunid [14]. However, the measurement was made using traditional direct (manual) anthropometry method. Their samples consisted of convenient sampling of 100 healthy 18–25 years-old men and women in equal number. Twenty-two linear measurements were performed and the result was compared to the Singaporean Chinese from Farkas’ study. They concluded that three features, namely the height of the head, intercanthal width and protrusion of the nasal tip may be useful to differentiate a Malay face from that of the Singaporean Chinese. The limitation of the study was that body mass index (BMI) was not considered in the inclusion criteria, which has been found to be correlated with facial muscle thickness [18]. In another study, Lin et al. [19] did a photogrammetric analysis to establish the nasolabial and mentolabial angles of Malaysian adults. However, they did not separate the subjects by the 3 main ethnic groups in Malaysia, which are the Malays (63.1%), Chinese (24.6%) and Indians (7.3%).

Knowledge of ethnic and gender specific normative data, including the mean and standard deviation of key facial measurements, is extremely useful in surgical procedures such as osteotomies, craniofacial corrections and reconstructive surgery [20]. These procedures can change the overlying soft tissues and subsequently the facial appearance. Thus raw baseline craniofacial anthropometric data of ethnicity, age and gender can lead to better clinical assessment and diagnosis. The data can be used to better plan surgical symmetry, predict post-surgical results, and compare pre-surgical goals with post-surgical results [21]. More effective and comfortable ergonomic products such as helmets, masks, eyeglasses and respirators can also be designed using the data [21]. It can also be valuable in forensic reconstructions and identification of missing persons [20].

To date, only one study has been done on soft tissue morphology of adult Malaysians of Malay ethnicity using stereophotogrammetry [22]. However, the study was focused only on the morphology of the nasolabial regions. To our knowledge, no previous anthropometric studies have been conducted on those of Malay ethnicity from neighbouring countries. There is one on Javanese females, using the photogrammetric method [23]. Therefore, the objectives of this study were to establish 3D facial soft tissue morphological values for Malay adults in Malaysia; and determine the morphological differences between genders using a non-invasive stereo-photogrammetry 3D camera.

## Materials and Methods

### Study design

This was a cross sectional study involving the collection and analysis of 3D images. Approval was obtained from the Faculty of Dentistry, University of Malaya Medical Ethics Committee, reference number DF CD 1305/0030(P).

### Sampling and sample selection

Subjects were Malaysian adults of Malay ethnicity. One hundred and nine subjects participated in this research, 54 Malay men (mean age: 22.4 [± 2.4]) and 55 Malay women (mean age: 23.2 [± 2.4]). The inclusion criteria were: 1) Malaysians of Malay ethnicity up to the second generation: The subjects verified their ethnicity via a self-administered questionnaire; 2) Age between 20 and 30 years; 3) Balanced and harmonious facial soft tissue profile. This included: (a) Class I skeletal relationship, which was assessed from the subjects’ profile, with the upper jaw slightly ahead of the lower by 2-4mm, (b) symmetrical face which was assessed by viewing the subject from the front and also looking down the face from behind and above, comparing the left and right sides of the face. In this assessment, the face was divided equally vertically from trichion to glabella, from glabella to subnasale and subnasale to chin. Facial symmetry was acceptable when the left and right proportions were not obviously different, and (c) competent lips, whereby the upper and lower lips were in contact at rest without requiring any muscle strain; 4) Class I incisor relationship with no adverse skeletal deviation; and 5) Healthy BMI for Asians (18.4–24.9 kg/ m^2^). Exclusion criteria were: 1) Subjects of mixed or uncertain ethnicity; 2) Patients with congenital dento-facial deformity or syndromic patients involving the craniofacial region; 3) Subjects who were undergoing or have undergone orthodontic treatment, orthognathic or facial plastic surgery; or other cosmetic facial aesthetic procedures; and 4) History of facial trauma.

The age range for other 3D imaging studies on facial anthropometry varies. Ngeow and Aljunid [14] studied craniofacial anthropometric norms of Malaysian Malays using traditional direct measurement methods, setting the age range at 18–25 years. They chose this age range based on the study by Hajnis et al. [24], which compared differences of the craniofacial complex between three racial groups: Chinese, Afro-Americans and North American Caucasians. A similar age range was chosen by Othman et al. [25] to assess the effects of primary and palatal repair after major growth has ceased. Farkas [26] reported that incremental changes in the nasal region continued after 18 years but at a slower rate. Torlakovic and Faerøvig [27] further noted that the nasal area did not show any significant changes between the ages of 20 and 30 years. Hence the age range of 20–30 years was selected for this study due to the stability of the soft tissue growth.

Subjects were recruited from around the Klang Valley. Each participant was clinically screened extra- and intra-orally at the Faculty of Dentistry, University of Malaya by LPM. Height and weight were measured to calculate their BMI, which is the body weight in kilograms divided by height in meters squared. The cut-off points for Asians (18.4–24.9 kg/ m^2^) were used as a guideline for this study [28, 29]. Healthy BMI was one of the inclusion criteria in this study, as facial soft tissue in an underweight or overweight person may influence measurements. For example, the thickness of the masseter muscle has been found to be correlated with BMI [18]. Since the study aimed to provide the soft tissue anthropometric norms, subjects with unhealthy BMI may confound the results. None of the previous studies had this criterion in their inclusion criteria. The World Health Organization (WHO) stated that generally Asians have a higher percentage of body fat than Caucasians of the same age, sex, and BMI. It recommended that the healthy BMI for Asian populations be 18.5–23 kg/m^2^, with a further suggestion that the cut-off point for observed risk in different Asian populations should vary from 22 kg/m^2^ to 25 kg/m^2^ [29]. According to the Malaysian Dietary Guidelines 2010 which was launched on 25 March 2010, in conjunction with the 25th Scientific Conference of the Nutrition Society of Malaysia, a healthy BMI for Asians is 18.4–24.9 kg/m^2^ [28]. Hence this figure was selected in our research. The BMI is intended to give a general sense of a healthy body weight. Nonetheless, BMI can be inaccurate for people who are fit or athletic, because their high muscle mass can list them as overweight, even though their body fat percentages are relatively healthy.

All suitable subjects were given verbal and written explanation and were invited to join the research. Written consent was obtained from all participants.

The power and sample size calculation was done using PS calculator software version 3.0.43, 2011 [30]. The sample size was determined from the parameters of the changes of the nose, which is normally unaffected by orthodontic treatment and shows the greatest variance [15]. With the likely change of 2 mm after the post growth period (>15 years) and a standard deviation of 2.8mm, a power of 0.90 and significance of 0.05 requires 42 samples [16]. Taking into consideration a possible 30% dropout rate, 55 subjects per gender were required. We had one male drop out.

### Image capture, measurement, and analysis of 3D images

3D photographs of all selected subjects were captured by LPM using the VECTRA-M5 360 (Canfield Scientific Inc, Fairfield, NJ, USA) camera, available in the 3D lab of the Dental Faculty at the University of Malaya for full-face imaging. Images were taken in a natural head position as described by Moorees and Keane [31]. The VECTRA-M5 360 camera consisted of five pod systems with a pair of camera lenses for every pod, producing 3D stereophotograms. These five pairs of identical cameras were separated by a known base distance. The 3D model image constructed from the fusion of ten images acquired by these ten lenses will give a 360 degrees imaging of the subject, to ensure image consistency and magnification. Prior to every imaging session, VECTRA M5-360 Head System calibration was performed according to the manufacturer’s instructions. This involved capturing and processing of an ‘L’ image on a white board. Stereophotogrammetry was used due to its reliable, safe and rapid performance in capturing images. In addition, these images can be stored and retrieved later to allow detailed or repeated measurements, or to compare pre- and post-treatment results. The no-touch measurement eliminates the need for direct contact, so it has no effect on surface pressure, which can influence the reliability of a measurement.

During image capture, the participant was seated on a self-adjustable stool with the head in a natural position [31]. Participants were instructed to look at a point ahead at eye level, with a neutral facial expression, the mandible in a resting position and the lips lightly opposed without undue muscular effort. The image had to be positioned precisely within the focus of the system, in the range of the five viewing panels. The image was then captured in 2 milliseconds under standard fluorescent lighting at normal lighting levels. It was then processed and converted to a 3D image within 2–3 minutes, on a desktop computer with a standard setting.

**Fig 1:**
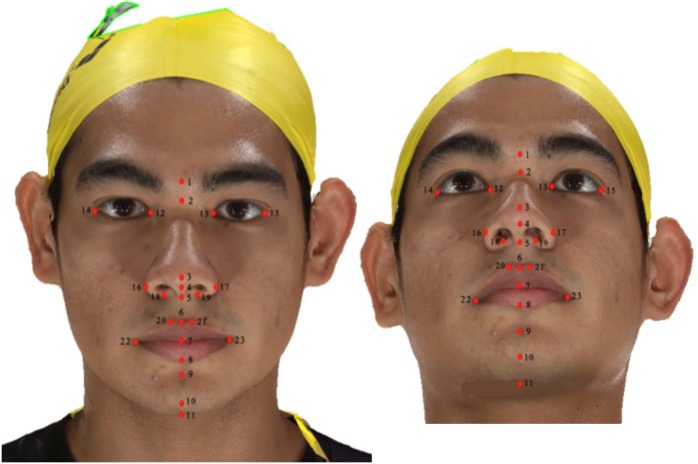
The digitized landmarks.

Soft tissue anthropometric landmarks related to the face, eyes, nose, orolabial, chin and throat areas, based on those suggested by Farkas [26], Othman et al. [8] and Baik et al [32], were identified and the points were recorded manually by LPM using Canfield Mirror imaging software (Table 1) (Fig 1). Seventeen linear and 3 angular measurements were computed automatically from the identified landmarks, while 2 facial ratios were derived from the linear measurements (Table 2).

**Table 1:** Landmarks and definitions.

Landmark	Definition		Landmarks in Fig 1
**Upper facial landmarks**			
*Glabella (g)	The most prominent point on the frontal bone in the midsagittal plane between the eyebrow ridges, just above the nose.		1
+Endocanthion (enR, enL)	The inner commisure point of the eye fissure		12, 13
+Exocanthion (exR, exL)	The outer commisure point of the eye fissure		14, 15
*Soft tissue nasion (n)	Deepest concavity point on the nasolabial suture, identical to the hard tissue nasion		2
**Nasolabial landmarks**			
+Alare (alR, alL)	The most lateral point on each alar contour		16, 17
+Subalare (sbalR, sbalL)	Junction between upper lip and alar base root		18, 19
*Pronasale (prn)	Most prominent midline point on the nose tip, identified on the lateral view		3
*Subnasale (sn)	Midline junction point between columella and upper lip		5
*Labrale superius (ls)	Midpoint of upper vermilion line		6
*Labrale inferius (li)	Midpoint of lower vermilion line		8
*Columellar high point (c)	Highest point on the columella crest		4
+Christa philtri (cphR, cphL)	Junction between upper lip vermilion and philtral peak		20, 21
*Stomion (sto)	Midpoint between upper and lower lip		7
+Cheilion (chR, chL)	The point where the outer edge of the upper and lower vermilions meet at the outer corner of the mouth		22, 23
**Lower facial landmarks**			
*Soft tissue pogonion (pog)		Most anterior midpoint of the chin	9
*Soft tissue gnathion (gn)		Most inferior midpoint of the chin	10
*Cervical point (cp)		Most inferior midpoint of the chin	11

*Midline

^+^ Bilateral (R right and L Left)

**Table 2:** Facial measurements and ratios.

Linear Measurements	
**Ocular dimensions**	
Biocular width	exR–exL
Ocular width right	exR–enR
Ocular width left	exL–enL
Intercanthal width	enR–enL
**Nasal dimension**	
Nasal tip protrusion	sn-prn
Nose width	al–al
Nose height	n-sn
Nasal bridge length	n-prn
Alar base root width	sbaR–sbaL
**Orolabial dimension**	
Upper lip length	sn–sto
Mouth width	ch-ch
Philtrum width	cph-cph
Philtrum length	sn-ls
Upper vermilion height	ls-sto
Lower vermilion height	sto-li
**Face measurement**	
Face height	n-gn
Lower Face height	sn-gn
**Angular Measurement**	
Facial profile angle	g-sn- pog
Nasolabial angle	ls-sn-col
Lower face- throat angle	cp -pog- sn
**Ratios**	
Nose width to mouth width	al–al / ch–ch
Lower facial height to total facial height	sn–gn / n–gn

### Method error assessment/analysis

The operator (LPM) was calibrated with an experienced research assistant in 3D facial assessment (RA) by measuring 10 images (5 men and 5 women). These 3D images were randomly picked using the covariate adaptive randomization technique available on www.randomization.com. For intra-examiner reliability analysis, the landmarks were digitized on the images and facial variables measured. Following the first measurements, the landmarks were not saved. After a two-week period, the landmarks and variables were re-digitized and measured again. For inter-examiner reliability assessment, measurements by LPM were compared to the measurements carried out by the RA. Both intra- and inte- examiner reliability tests were analyzed using intra-class correlation coefficient (ICC). The ICC scores for intra-examiners were found to be in good agreement, ranging from 0.827–0.987. Meanwhile, the ICC scores for inter-examiners were found to be between acceptable to good agreement, ranging from 0.700 (ocular width right) to 0.983 (nasolabial angle) (S1 Table).

The poorest reliability was noticed in the inter-observer assessment for right ocular width. Poor reproducibility observed in association with the eyes was because this area was difficult to capture using a laser-based acquisition system; which may affect mesh production during computerized processing of the 3D facial images [27]. The complex geometry associated with the eye region could also be the reason for it being the least reproducible point [8].

### Statistical analysis

Data collected were analyzed using Statistical Package for the Social Sciences (SPSS) version 12.0. Initial analysis showed a mixture of normally and non-normally distributed data for the dependent variables. The data was then log-transformed (log_10_). The transformed data was confirmed to be normally distributed by histograms and Q-Q plots; hence was used in the parametric analysis. Mean and standard deviation were used to establish the Malay facial morphological values based on the original data. A MANOVA was carried out using the log-transformed data to investigate differences between genders as the independent variable, and the variables of interest as the dependent variables. Post-hoc Bonferroni adjustment was also performed to avoid Type 1 error. The initial level of significance was set at *p* < 0.05 and the adjusted statistical value after Bonferroni correction, taking into account the 22 dependent variables, was *p* < 0.0023. For clinically significant differences, a cut-off minimum value of 3mm was set, based on a proposal that differences of up to 2mm between the two hemi-faces were considered to be within the normal range [26]. For angular measurement, no previous studies have suggested a threshold for clinically relevant differences expressed in degrees. Therefore, a threshold of 10 degrees was decided to be clinically significant by our panel of experts. A difference of less than 10 degrees would not be visually discernible to the untrained eye (S2 Table).

## Results

The MANOVA analysis showed statistically significant differences (p < 0.0023) between genders for the variables concerned. Table 3 demonstrates the mean, standard deviation and mean differences, as well as the comparison of the variables between men and women by MANOVA analysis. In general, the Malay men when compared to women demonstrated larger statistically significant differences in almost all parameters of the craniofacial region, except for the intercanthal widths, upper and lower vermillion heights, facial profile and nasolabial angles, and in the nose to mouth and lower face height to total face height ratios.

**Table 3:** Mean, standard deviation, mean differences and *p*-value of facial morphologic value differences between Malay male and female.

Measurements	Malay					
	Male (n = 54)	Female (n = 55)	Mean difference (95% CI)	^∞^F statistics df	^∞^*p*- value	^∞^*Partial η*^*2*^
	Mean (SD)	Mean (SD)				
**Ocular dimension**						
Biocular width (mm)	96.19 (4.64)	92.05 (3.22)	4.14^#^ (2.64–5.64)	29.40 (1)	0.00000*	0.22
Right ocular width (mm)	30.89 (2.22)	29.38 (1.71)	1.51 (0.76–2.26)	15.80 (1)	0.00013*	0.13
Left ocular width (mm)	31.25 (2.01)	29.48 (1.89)	1.77 (1.03–2.51)	22.43 (1)	0.00001*	0.17
Intercanthal width (mm)	35.74 (2.79)	35.03 (2.47)	0.71 (-0.29–1.71)	1.96 (1)	0.16476	0.02
**Nasal dimension**						
Nasal tip protrusion (mm)	17.26 (1.78)	16.11 (1.74)	1.15 (0.48–1.81)	11.53 (1)	0.00096*	0.10
Nose width (mm)	39.59 (2.25)	36.67 (2.40)	2.92 (2.04–3.80)	42.82 (1)	0.00000*	0.29
Nose height (mm)	54.13 (3.61)	49.20 (3.48)	4.93^#^ (3.59–6.27)	52.98 (1)	0.00000*	0.33
Nasal bridge length (mm)	46.86 (3.29)	41.13 (6.60)	5.73^#^ (3.75–7.72)	32.74 (1)	0.00000*	0.23
Alar base root (mm)	18.58 (2.10)	17.28 (1.82)	1.30 (0.56–2.04)	12.01 (1)	0.00077*	0.10
**Orolabial dimension**						
Upper lip length (mm)	22.77 (2.12)	21.31 (1.76)	1.46 (0.72–2.20)	15.26 (1)	0.00016*	0.13
Mouth width (mm)	50.83 (3.75)	48.00 (2.61)	2.83 (1.61–4.06)	20.99 (1)	0.00001*	0.16
Philtrum width (mm)	11.84 (1.90)	10.40 (1.14)	1.44 (0.80–2.08)	20.19 (1)	0.00002*	0.16
Philtrum length (mm)	14.19 (2.31)	12.71 (1.95)	1.47 (0.66–2.29)	12.94 (1)	0.00049*	0.11
Upper vermillion height (mm)	10.30 (1.91)	10.30 (1.61)	0.00 (-0.68–0.66)	0.00 (1)	0.98528	0.00
Lower vermillion height (mm)	10.23 (2.01)	9.82 (1.69)	0.41 (-0.30–1.12)	1.34 (1)	0.24949	0.01
**Face measurement**						
Face height (mm)	119.89 (5.69)	111.58 (5.22)	8.31^#^ (6.24–10.38)	63.12 (1)	0.00000*	0.37
LFH (mm)	68.65 (4.58)	64.73 (4.23)	3.92^#^ (2.24–5.59)	21.52 (1)	0.00001*	0.17
**Angular measurement**						
Facial profile angle (X°)	166.91 (5.21)	169.52 (4.60)	-2.61 (-4.47– -0.74)	7.67 (1)	0.00662	0.07
Nasolabial angle (X°)	105.06 (9.60)	101.90 (9.33)	3.16 (-0.43–6.75)	3.04 (1)	0.08412	0.03
Lower face throat angle (X°)	116.94 (6.70)	111.72 (6.46)	5.22 (2.72–7.72)	17.11 (1)	0.00007*	0.14
**Ratio measurement**						
Nose: mouth width (ratio)	0.75 (0.06)	0.77 (0.06)	0.02 (-0.01–0.04)	2.14 (1)	0.14651	0.02
LFH:TFH (ratio)	0.57 (0.03)	0.58 (0.03)	-0.01 (-0.02-.003)	1.85 (1)	0.17633	0.02

^∞^MANOVA based on log-transformed data

*statistical significance value after Bonferroni adjustment: *p* < 0.0023;

^#^clinically significant > 3mm

In terms of clinically significant differences (mean difference >3mm), in the eye region, the biocular width was significantly wider in Malay men compared to women (mean difference 4.14mm). The male nose was generally longer and more prominent. Nose height and nasal bridge length were clinically larger significantly, with mean differences of 4.93mm and 5.73mm respectively. Malay men had longer faces as demonstrated by the increase in face height (mean difference 8.31mm) and in the lower face height (mean difference 3.92mm).

Four variables in the orolabial dimensions were found to be significantly larger statistically (p < 0.0023) in men but the differences were not clinically significant (mean differences between 1.44 to 2.83mm). There was only 1 variable in the angular measurements that was significantly different. Malay men also had wider lower face throat angle but the difference was not clinically significant (5.21 degrees).

In summary, statistically and clinically relevant findings between Malay men and women were only noted for the biocular width, nose height, nasal bridge length, face height and lower face height.

## Discussion

Young Malay adults were chosen for this study because they are from the main ethnic group in Malaysia and patients who are assessed for pre-surgical orthognathic treatment are generally young adults. Thus, this study may provide anthropometric information for surgical planning for this group. Due to limited resources, random sampling of the Malaysian population was not feasible. Participants originated from all parts of Malaysia and were currently studying or working in Kuala Lumpur, the capital city of Malaysia. Hence, within the limitations of this study, the results derived provide preliminary data that may be used to represent the Malay normal values. A larger prevalence study of random samples around Malaysia is recommended for a more comprehensive representation of this group.

### Establishing normative data on facial morphology and comparing data to previous studies

One of the objectives of this study was to establish 3D facial soft tissue morphological values for the Malay ethnic group, using a non-invasive stereophotogrammetry 3D camera. There are three studies that have been done on the same ethnic group. However, one study used the direct traditional method and only measured 22 linear measurements [14]. The other studies employed the same 3D stereophotogrammetric method [22] as the present study; and the 2D photogrammetric method [19], but both only focused on the nose and mouth regions. Normative anthropometric facial morphology data of adult Malaysians of Malay ethnicity are tabulated in Table 4; with comparison data from Ngeow and Aljunid [14] and Al-Khatib et al. [22]. It can be observed that the normative data values can be generalized as being within the same range. The apparent differences were noted in the biocular width in men, and nose height and face height in females, which could be due to dissimilarity in methodology and the slightly younger age group of the subjects in Ngeow and Aljunid [14].

**Table 4:** Comparison of anthropometric facial morphologic norms of the Malays between Ngeow and Aljunid [14], Al-Khatib et al. [22] and the present study.

Measurements	Male				Female		
	Mean (SD)				Mean (SD)		
	Present study (n = 54)	Ngeow and Aljunid [15] (n = 50)		Al-Khatib et al. [20] (n = 50)	Present study (n = 55)	Ngeow and Aljunid [15] (n = 50)	Al-Khatib et al. [20] (n = 50)
**Ocular dimension**							
Biocular width (mm)	96.19 (4.64)	92.3 (4.1)		-	92.05 (3.22)	89.6 (3.2)	-
Right ocular width (mm)	30.89 (2.22)	-		-	29.38 (1.71)	-	-
Left ocular width (mm)	31.25 (2.01)	29.5 (1.5)		-	29.48 (1.89)	28.7 (1.4)	-
Intercanthal width (mm)	35.74 (2.79)	33.9 (1.9)		-	35.03 (2.47)	32.5 (1.7)	-
**Nasal dimension**							
Nasal tip protrusion (mm)	17.26 (1.78)	18.1 (1.6)		-	16.11 (1.74)	17.5 (1.5)	-
Nose width (mm)	39.59 (2.25)	41.0 (2.0)		-	36.67 (2.40)	37.3 (2.6)	-
Nose height (mm)	54.13 (3.61)	51.6 (3.5)		-	49.20 (3.48)	54.1 (2.9)	-
Nasal bridge length (mm)	46.86 (3.29)	-		-	41.13 (6.60)	-	-
Alar base root (mm)	18.58 (2.10)	-		-	17.28 (1.82)	-	-
**Orolabial dimension**							
Upper lip length (mm)	22.77 (2.12)	22.7 (2.0)		-	21.31 (1.76)	21.1 (1.9)	-
Mouth width (mm)	50.83 (3.75)	48.8 (3.5)		-	48.00 (2.61)	47.1 (3.5)	-
Philtrum width (mm)	11.84 (1.90)	-		-	10.40 (1.14)	-	-
Philtrum length (mm)	14.19 (2.31)	13.1 (1.7)		-	12.71 (1.95)	12.2 (1.8)	-
Upper vermillion height (mm)	10.30 (1.91)	9.8 (1.1)		-	10.30 (1.61)	9.1 (1.0)	-
Lower vermillion height (mm)	10.23 (2.01)	12.0 (1.6)		-	9.82 (1.69)	11.0 (1.2)	-
**Face measurement**							
Face height (mm)	119.89 (5.69)		119.3 (6.2)	-	111.58 (5.22)	118 (5.8)	-
LFH (mm)	68.65 (4.58)		68.5 (5.2)	-	64.73 (4.23)	63.2 (4.7)	-
**Nasal dimension**							
Nasal tip protrusion (mm)	17.26 (1.78)		-	18.44 (3.23)	16.11 (1.74)	-	17.69 (12.29)
Nose width (mm)	39.59 (2.25)		-	40.27 (2.22)	36.67 (2.40)	-	37.43 (2.39)
Nose height (mm)	54.13 (3.61)		-	56.04 (4.62)	49.20 (3.48)	-	51.24 (3.55)
Nasal bridge length (mm)	46.86 (3.29)		-	45.91 (4.96)	41.93 (3.46)	-	41.95 (4.14)
Alar base root (mm)	18.58 (2.10)		-	23.11 (2.44)	17.28 (1.82)	-	22.12 (2.40)
**Orolabial dimension**							
Upper lip length (mm)	22.77 (2.12)		-	23.42 (2.45)	21.31 (1.76)	-	20.96 (1.94)
Mouth width (mm)	50.83 (3.75)		-	51.26 (3.40)	48.00 (2.61)	-	49.38 (3.99)
Philtrum width (mm)	11.84 (1.90)		-	13.11 (2.31)	10.40 (1.14)	-	12.32 (2.10)
Philtrum length (mm)	14.19 (2.31)		-	13.81 (2.69)	12.71 (1.95)	-	13.02 (1.99)
Upper vermillion height (mm)	10.30 (1.91)		-	10.32 (1.88)	10.30 (1.61)	-	9.16 (1.65)
Lower vermillion height (mm)	10.23 (2.01)		-	9.87 (2.34)	9.82 (1.69)	-	10.08 (2.12)

Al-Khatib et al. [22], used stereophotogrammetry to measure 11 parameters of the nasolabial area. Their samples were divided into 3 age groups; 13–14, 15–17 and 18–36 years. Only the 18–36 years age group data was compared because it is the closest age match to the present sample. Most of the measurements from the present study were slightly less than their study, with apparent narrower alar base root length in both men and women. In addition to the slight age group discrepancy of the sample used, the differences could also be confounded by the anatomy of the nose as landmark identification is usually more challenging at curvatures.

### Sex differences in facial morphology

Assessment of sexual dimorphism is an essential component of anthropometry, hence the second objective of this study was to determine the morphologic differences between genders. Gender differences have been reported in the literature and therefore it is clinically important to treat patients according to the gender norms. In general, Malay men were observed to demonstrate larger readings in almost all parameters in the facial region. The facial profile angle was slightly more acute in men but the difference was not significant. The significant differences observed in almost all parameters concurred with the majority of findings by other studies although the variables may differ [1, 12, 14, 33]. It has to be stressed that comparisons to other studies should be interpreted with caution as the differences in study design, measurement protocols and statistical analyses prevent direct comparison of this research result to other direct anthropometry method and 3D digital photogrammetry systems [1, 12, 15, 16] and also to other local facial anthropometry studies [14, 19, 22].

Only the biocular width (4.14mm) in the eye region, nose height (4.93mm) and nasal bridge length (5.74mm) in the nasal region, face height (8.31mm), and lower face height (3.92mm) were statistically and clinically wider significantly in Malay men compared to Malay women in the current study. It was also observed that the right ocular width and left ocular width were statistically different significantly but not clinically because the mean differences were less than 3mm (mean differences 1.51mm and 1.77mm respectively). This contradicted a previous report that showed no significant differences of the eyes in ethnic Malays [15]. The differences might be because of the traditional method employed in the other study. Direct measurement with a calliper was used; hence taking measurements around the eye area was difficult due to the risk of injuring the eyes. Therefore, they might have placed the point slightly away from the eyes which may have confounded their study and produced different results from the current study.

In the nasal region, all parameters were significantly different showing that generally the male nose is longer and more prominent. Only the nose height and nasal bridge length demonstrated clinically significant difference, with mean difference of 4.93mm and 5.72mm, respectively. Ngeow and Aljunid [15] too had the same findings for nose width, height and nasal tip protrusion. Similarly, Al Khatib et al. [22] found that the nose width, nose height and nasal bridge length are significantly different between genders and suggested that it was related to the broader nasal areas, as well as to the larger and more prominent lips in males. They did not find gender dimorphism for nasal tip protrusion and alar base root width and this is similar to the findings in this research. These findings were also similar to other studies done in different ethnic groups. Baik et al. [12] compared linear, angular and facial depth ratio of Korean men and women in their 20’s. They observed that the nose is located more anteriorly and inferiorly in men and that the nose in males is longer and more prominent than in females. The philtrum width was also larger in men. Meanwhile, Dong et al. [16] established normative 3D facial models of 20–27 years old ethnic Han of central China by getting mean coordinates of facial landmarks. The linear models were then converted to polyhedron models and they compared the facial models of men and women. They found that the female model was smaller in lateral and vertical directions and was also less developed in the anteroposterior direction. Gor et al. [34] assessed the use of 3D facial averages in determining facial morphological differences in white populations of Budapest and Houston. Although they used a more sophisticated combination of complex algorithms, they also observed that generally men had larger noses. Larger noses in men as found in this study and also previous studies could be influenced by higher body mass in men and associated oxygen consumption demands required for tissue maintenance [35]. Males are also characterized by absolutely and relatively larger internal nasal cavities due, in part, to relatively and absolutely taller nasal airways [36, 37].

Interestingly, we did not find any clinically significant gender dimorphism in the orolabial region. However, the men showed larger readings for upper lip length (mean difference: 1.45mm), mouth width (mean difference: 2.83mm), philtrum width (mean difference: 1.44mm), and philtrum length (mean difference: 1.47mm). Ngeow and Aljunid [14] found all the parameters to be statistically different, but clinically all the mean differences were less than 3mm, similar to our findings. According to Al- Khatib et al. [22] the upper lip length was found to be statistically different significantly showing that the men have a longer upper lip length compared to women, but their finding was also not clinically significant.

No clinically significant differences were observed between both genders for the angular and ratio measurements. The nasolabial angle in the Malay sample was found not to be clinically gender dimorphic and this finding is in agreement with Lin et al. [19]. They analyzed the nasolabial and mentolabial angles norm in Malaysian adults using a photogrammetric method and found that there was no significant gender difference. However, direct comparisons must be interpreted with caution as they were using a 2D method and their sample was a mix of the three main ethnic groups in Malaysia. An interesting finding observed is the facial profile angle was the only parameter showing larger reading for women compared to men. However, with the mean difference of only 2.62 degrees, this makes it clinically insignificant.

The face measurement presented by face height and lower face height exhibited clinically significant differences between the genders (mean differences 8.30mm and 3.92mm respectively). Male faces were observed to be longer than women’s. These two measurements concur with the findings by another local study [14] and also with a study done on Korean adults [12]. However, the result obtained was contradictory to that of a study done on healthy Turkish young adults using a photographic 2D method [38]. They did not find any clinically significant differences in the gender for the face height and the lower face height.

Genetic and environmental factors too might have contributed to the differences observed in facial soft tissue dimensions within and between human populations [39]. The morphological variations noted in different populations may due to the proportion and type of genetic control that varies between individuals and groups. Hence, genetic factors have been proposed to exert substantial influence on the variation observed in the shape and configuration of the human face [40–42]. Environmental factors such as climate could influence facial height and width as well as nasal height and cranial width, and thus may be a contributing factor to the differences in facial features among populations [43, 44].

## Conclusions

The facial soft tissue morphological values of adult Malay men and women in Malaysia have been analyzed and average data established in this research using a non-invasive stereo-photogrammetry 3D camera. Generally, all facial measurements analyzed were larger in males. Clinically significant gender dimorphisms existed in biocular width, nose width, nasal bridge length, face height and lower face height values. These can be interpreted to mean that Malaysian Malay men, compared to women, have a wider distance between the eyes, longer and more prominent noses and longer faces. The quantitative data obtained from this study can be used in forensic identification of corpses, criminals and missing persons. It also could probably be utilized by designers and manufacturers of ergonomic personal protective equipment that conforms to the facial structure of the local population. In the clinical setting, the average values can facilitate comprehensive medical and dental diagnosis of the facial region for planning purposes and to evaluate the progress and the result of treatment. The data from this study could be expanded to suggest a 3D coordinate system and measurement items for the 3D analysis of Malaysian Malay facial soft tissue, to acquire averaged values of these items, and subsequently to produce facial templates.

## Supporting Information

S1 Table ICC of intra and inter examiners reliability test. (DOCX)

S2 Table Malay data. (XLSX)
