# Supplementary material for: Anthropometric Study of Three-Dimensional Facial Morphology in Malay Adults
Source: PLoS One. 2016 Oct 5;11(10):e0164180. doi: 10.1371/journal.pone.0164180 (PMC5051712; doi:10.1371/journal.pone.0164180)
Supplement: S1 Table — (DOCX) [file pone.0164180.s001.docx]

S1 Table. ICC of intra and inter examiners reliability test

| **Parameters** | **ICC** | |
| --- | --- | --- |
|  | **Intra-examiner** | **Inter-examiner** |
| **Ocular dimension** | | |
| Biocular width | 0.987 | 0.938 |
| Ocular width right | 0.891 | 0.700 |
| Ocular width left | 0.864 | 0.850 |
| Intercanthal width | 0.869 | 0.807 |
| **Nasal dimension** | | |
| Nasal tip protrusion | 0.925 | 0.901 |
| Nose width | 0.985 | 0.971 |
| Nose height | 0.827 | 0.737 |
| Nasal bridge length | 0.849 | 0.871 |
| Alar base root width | 0.953 | 0.952 |
| **Orolabial dimension** | | |
| Upper lip length | 0.928 | 0.923 |
| Mouth width | 0.958 | 0.902 |
| Philtrum width | 0.867 | 0.969 |
| Philtrum length | 0.913 | 0.918 |
| Upper vermilion height | 0.876 | 0.814 |
| Lower vermilion height | 0.848 | 0.912 |
| **Face measurement** | | |
| Face height | 0.885 | 0.977 |
| Lower Face height | 0.924 | 0.978 |
| **Angular measurement** | | |
| Facial profile angle | 0.957 | 0.951 |
| Nasolabial angle | 0.972 | 0.983 |
| Lower face- throat angle | 0.865 | 0.908 |
